# *Lactobacillus acidophilus* Mitigates Osteoarthritis-Associated Pain, Cartilage Disintegration and Gut Microbiota Dysbiosis in an Experimental Murine OA Model

**DOI:** 10.3390/biomedicines10061298

**Published:** 2022-06-01

**Authors:** InSug O-Sullivan, Arivarasu Natarajan Anbazhagan, Gurjit Singh, Kaige Ma, Stefan J. Green, Megha Singhal, Jun Wang, Anoop Kumar, Pradeep K. Dudeja, Terry G. Unterman, Gina Votta-Velis, Benjamin Bruce, Andre J. van Wijnen, Hee-Jeong Im

**Affiliations:** 1 Department of Medicine, University of Illinois Chicago, Chicago, IL 60612, USA; insug@uic.edu (I.O.-S.); arivu29@uic.edu (A.N.A.); meghasinghal0107@gmail.com (M.S.); anoop@uic.edu (A.K.); pkdudeja@uic.edu (P.K.D.); unterman@uic.edu (T.G.U.); 2 Biomedical Engineering, University of Illinois Chicago, Chicago, IL 60612, USA; gurjitfauji@gmail.com (G.S.); makaige225@gmail.com (K.M.); junwang602@gmail.com (J.W.); 3 Genomic Research Core, Research Resources Center, University of Illinois Chicago, Chicago, IL 60612, USA; greendna@uic.edu; 4 Jesse Brown Veterans Affairs Medical Center, Chicago, IL 60612, USA; ginavot@gmail.com (G.V.-V.); benmed@gmail.com (B.B.); 5 Anesthesiology, University of Illinois Chicago, Chicago, IL 60612, USA; 6 Biochemistry, University of Vermont, Burlington, VT 05405, USA

**Keywords:** osteoarthritis, probiotics, OADMD, dysbiosis

## Abstract

To test probiotic therapy for osteoarthritis (OA), we administered *Lactobacillus acidophilus* (LA) by oral gavage (2×/week) after induction of OA by partial medial meniscectomy (PMM). Pain was assessed by von Frey filament and hot plate testing. Joint pathology and pain markers were comprehensively analyzed in knee joints, spinal cords, dorsal root ganglia and distal colon by Safranin O/fast green staining, immunofluorescence microscopy and RT-qPCR. LA acutely reduced inflammatory knee joint pain and prevented further OA progression. The therapeutic efficacy of LA was supported by a significant reduction of cartilage-degrading enzymes, pain markers and inflammatory factors in the tissues we examined. This finding suggests a likely clinical effect of LA on OA. The effect of LA treatment on the fecal microbiome was assessed by 16S rRNA gene amplicon sequencing analysis. LA significantly altered the fecal microbiota compared to vehicle-treated mice (PERMANOVA *p* < 0.009). Our pre-clinical OA animal model revealed significant OA disease modifying effects of LA as reflected by rapid joint pain reduction, cartilage protection, and reversal of dysbiosis. Our findings suggest that LA treatment has beneficial systemic effects that can potentially be developed as a safe OA disease-modifying drug (OADMD).

## 1. Introduction

Osteoarthritis (OA) causes chronic pain due to progressive articular cartilage degradation, synovial inflammation and structural changes in subchondral bone morphology. Clinically, OA joints display joint line tenderness, synovial hypertrophy and increased inflammatory cytokines and metalloproteinases [1]. OA becomes more prevalent with age and obesity and up to 35% of people over 60 years of age suffer from OA [2]. Current treatment options for OA are neither effective nor curative, and some drugs may cause drug dependence and imbalances in the natural microflora of the gut [3]. Therefore, it is critical to develop new, effective and safe therapies for OA treatment.

Recent human and mouse studies have demonstrated changes in gut microbiome in OA [4]. Reduced alpha diversity and altered functional composition of the gut microbiome is suggested to occur in OA. In human subjects, the order Clostridiales correlates negatively with OA and genus Streptococcus associates with OA pain [4]. Metabolomic studies on fecal samples from human OA subjects have revealed associations of leukotriene and amino acid metabolism, glycosylphosphatidylinositol anchor biosynthesis, and pyruvate metabolism with OA reflecting functional changes in the gut microbiome in OA [5]. Similar but more robust observations have been made in mice. Depletion of the gut microbiome in gnotobiotic or antibiotic treated mice has been shown to alleviate OA in different animal models of the disease [4,6,7]. Furthermore, germ-free mice colonized with the fecal microbiome of human OA subjects exhibit higher OA severity in a surgical OA model [4,8]. These data suggest that gut microbial communities contribute to the pathology of OA.

The OA-associated microbial imbalance (dysbiosis) [9] can potentially be rectified by reintroducing specific and non-pathogenic bacteria and yeast (probiotics), which reduce systemic inflammation [10]. Oral microbiome and probiotic applications to improve bone health and OA have been extensively considered [11,12,13]. *Lactobacillus* species represent well-characterized probiotics with established antimicrobial and anti-inflammatory effects [14]. *Lactobacillus acidophilus* (LA) has also been shown to ameliorate pain and prevent cartilage destruction in a chemically induced OA rat model [15]. Gut barrier dysfunction has also been reported in OA [3]; however, it remains to be established whether *Lactobacillus* species can modulate gut inflammation and restore the gut microbiome during OA treatment. Therefore, we pursued a probiotic approach using LA to reduce OA pain and preserve joint integrity. This probiotic therapy complements other emerging treatment strategies that target vascular endothelial growth factor (VEGF) and its cognate receptor signaling pathways for OA joint pain and inflammation [16]. Here, we report the biological effects of LA that was orally administered at the time of joint injury in our preclinical OA animal model, suggesting a strong role for the gastrointestinal microbiota in improved pathology.

## 2. Materials and Methods

### 2.1. Surgical Induction of Murine OA and Probiotic Treatment

Female C57BL/6 mice were purchased from Jackson Laboratories at 8–9 weeks of age and used for OA induction at 11 weeks of age followed by pain tests and oral administration of probiotics. Mice were maintained in groups of 2 to 4 mice at the Jesse Brown Veterans Affairs Medical Center (JBVAMC) Veterinary Medical Unit, an AAALAC accredited, specific pathogen-free rodent barrier facility in Chicago, IL, USA. Each experimental group encompassed 8 mice unless indicated otherwise.

**Figure 1:**
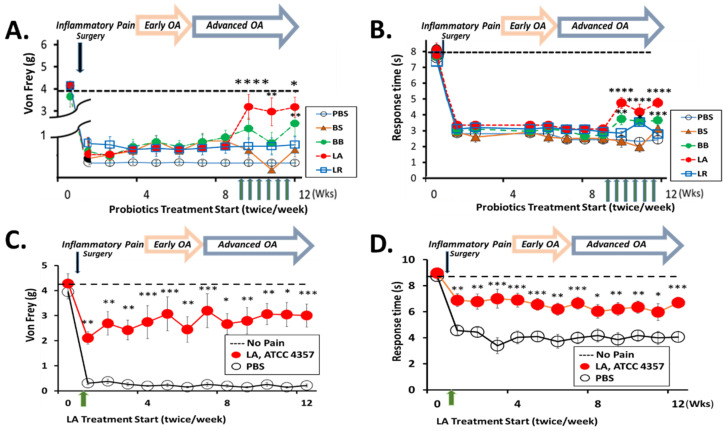
Effect of probiotics administered in advanced OA stage on joint pain and the disease-modifying effects of LA in our preclinical OA animal model (**A**,**B**). We administered 3 × 10^9^ colony-forming units (CFU) in 200 μL of *Lactobacillus acidophilus* (LA, ATCC 4356), *Bifidobacterium breve* (BB, ATCC 15700), *Lactobacillus reuteri* (LR, ATCC 55730) and *Bacillus subtilis* (BS, ATCC 6633) along with PBS to C57BL/6 mice (10–11 weeks old) by oral gavage 2 times/week starting at 9 weeks after PMM surgery. Behavioral pain responses were assessed weekly. Mechanical allodynia was by von Frey (**A**) and hot plate (**B**) tests. Number of animals/group: N = 8 each for PBS, BS, BB, LA and LR group. Effect of probiotics administered in inflammatory pain stage on joint pain and the disease modifying effects of LA in our preclinical OA animal model (**C**,**D**). Oral gavage of 3 × 10^9^ CFU/200 μL of LA (a single strain) was given twice/week, starting at the time of injury (inflammatory knee joint pain stage). The analgesic effect of LA was measured by weekly pain tests: mechanical allodynia by von Frey (**C**) and hot plate (**D**) tests. PBS treatments (black empty circles N = 4) were included as a vehicle control to compare with LA treatments (red filled circles N = 6). Number of animals/group: N = 8 each for (**A**,**B**) N = 4, 6 for (**C**,**D**). Data are expressed as mean ± S.E.M. Statistical analyses were carried out using 2-way ANOVA followed by Tukey’s multiple comparison analysis. The vehicle treatment group was compared with the probiotic group. Significance: * *p* < 0.05, ** *p* < 0.01 and *** *p* < 0.001, **** *p* < 0.0001 to PBS.

*Lactobacillus acidophilus* (LA, ATCC 4356), *Bifidobacterium breve* (BB, ATCC 15700), *Lactobacillus reuteri* (LR, ATCC 55730) and *Bacillus subtilis* (BS, ATCC 6633) were purchased from ATCC (Manassas, VA, USA). Cultures were grown overnight in MRS broth at 37 °C without shaking as we previously described [17]. OA was induced by partial medial meniscectomy (PMM) in 11-week-old female C57BL/6 mice (Jackson Laboratories) as described [18]. Mice were pre-treated with buprenorphine (0.1 mg/kg, sc) and anesthetized with ketamine (100 mg/kg, ip) and xylazine (5 mg/kg, ip) for PMM on the left hind knee. Body temperature was maintained using a heated surgical board and warming pad. Mice received twice/week oral gavage of 3 × 10^9^ CFU/200 μL probiotics or vehicle from 9 weeks to 12 weeks for advanced OA stage screening (Figure 1A,B). In subsequent studies, LA was administered two times a week starting at the time of injury (Figure 1C,D). Mice were subjected to pain testing weekly, and then were CO_2_-euthanized followed by tissue harvesting at 12 weeks post PMM surgery. Mice within the experimental group for Vehicle + Sham mice were treated with 200 μL PBS by oral gavage two times per week after sham surgery. Vehicle + OA group received 200 μL PBS by oral gavage two times per week after OA induced by PMM surgery. The LA + OA group received twice/week oral gavage of 3 × 10^9^ CFU/200 μL of LA after OA induced by PMM surgery. Mice were grouped together four per cage. Mice were housed at 25  ±  0.5 °C with a 12 h light–dark cycle at JBVAMC. All animal studies were approved by the Animal Care Committee of the UIC and JBVAMC (IACUC # 18-13, approved on 25 October 2018).

### 2.2. Longitudinal Behavioral Pain Measurements

Longitudinal pain was analyzed weekly by von Frey filaments (mechanical allodynia) [19] and hotplate testing until mice were 12 weeks post-PMM. Measurements were compared to baseline (obtained before OA induction). The threshold force required to elicit withdrawal of the paw was determined using a set of calibrated von Frey fibers (Stoelting Touch Test Sensory Evaluator Kit, Wood Dale, IL, USA). Filaments were applied to the plantar surface of the hind paw of mice placed on a metal grid floor in small plexiglass cubicles. Hind paw thermal hyperalgesia was measured using a Hotplate Analgesia Meter (Columbus Instruments, Columbus, OH, USA). Mice were placed onto a 55 °C hot plate, and timed for behavioral events (licking, hind paw shaking, and jumping). A cutoff latency of 30 s was used to prevent tissue damage [20].

### 2.3. Histopathology and Immuno-Fluorescence Microscopy

Histological and immuno-histochemical analyses [19] and gross knee-joint pathology were performed as previously described [21]. OA grading of decalcified knee joints was histologically evaluated using Osteoarthritis Research Society International (OARSI) scoring [22] in serial sagittal sections stained with Safranin-O/fast green. Protein markers discussed in the main text were analyzed by fluorescence microscopy (Nikon Eclipse NiE, Nikon Instruments Inc., Melville, NY, USA) in 4% paraformaldehyde-fixed, paraffin embedded sections of knee joints, distal colon (DC), lumbar (L4-5) spinal cord (SC) and DRG using primary antibodies against the proteins of interest and fluorophore-conjugated secondary antibodies and counterstained with the blue fluorescence of 4′,6-diamidino-2-phenylindole (DAPI).

### 2.4. Reverse Transcription and qPCR Analysis

Total RNA from distal colon was isolated (Qiagen RNA mini kit, Carlsbad, CA, USA), reverse transcribed (iScript™ cDNA Synthesis Kit, BioRad, Hercules, CA, USA) and analyzed by RT-qPCR (CFX Connect system, Bio-Rad Laboratories, Hercules, CA, USA) using the SYBR Green method (iQ™ SYBR^®^ Green Supermix Bio-Rad Laboratories). Relative mRNA expression was determined by the ΔΔCt method using β-Actin as control. Primer sequences for RT-qPCR were as follows: VEGFR1 (*Vegfr1*);F-TGGCCACCACTCAAGATTAC, R-TATAGACACCCTCATCCTCCTC, ARTN (*Artn*); F-CTCAGTCTCCTCAGCCCG, R-TCCACGGTCCTCCAGGTG, TNF-α(*Tfn*); F-TATGAGCCCATATACCTGGGAGGA, R-TCCCTTCACAGAGCAATGACTCCA, VEGFA (*Vegfa*); F-CCTGGCCCTCAAGTACACCTT, R-TCCGTACGACGCATTTCYAG, TrkA (*Ntrk1*); F-CTCCGTCATGGCTGCTTT, R-AACAGCACATCAAGAGACCC, GFRα-3 (*Gfra3*); F-ATCCTTGGGACTTGTGCAAC, R-GGGAGAAGAGCTGTCTGTGG, β-Actin (*Actb*); F-ACGATGCTCCCCGGGCTGTATT, R-TCTTGCTCTGGGCCTCGTCA.

### 2.5. Microbiome Analysis

DNA extracted from feces using Quick-DNA Fecal/Soil Microbe Microprep (Zymo Research) was two-step PCR amplified with primers targeting the V4 variable region of the microbial 16S rRNA gene as described [23]. Amplicons were sequenced (Illumina MiniSeq, San Diego, CA, USA) and reads processed through the DADA2 pipeline from the Research Informatics Core (RIC) at the University of Illinois at Chicago (UIC). Statistics and visualizations were performed using the Microbiome Analyst package (RIC, UIC).

### 2.6. Statistical Analyses

Statistical significance was determined using Student’s *t*-test or analysis of variance (ANOVA) for repeated measures, followed by 2-way analysis of variance ANOVA followed by Tukey’s multiple comparison analysis using GraphPad Prism software version 9.0.2. (Graphpad Software, San Diego, CA, USA). Data are expressed as mean ± S.E.M., and *p*-values < 0.05 were considered significant.

## 3. Results

### 3.1. Oral Administration of LA Relieves Knee OA Pain

In our search for probiotics having a specific efficacy against OA pain and joint pathology, we compared the pain reduction potential of four probiotics in advanced stages of surgically induced OA mice (N = 8 each for four probiotics and one PBS control vehicle group). Bacillus, Lactobacillus, and Bifidobacterium strains that have beneficial effects on inflammatory and autoimmune diseases were tested weekly for joint pain relief in advanced stages of OA. Among BS, BB, LA, and LR probiotics tested, LA treatments showed the fastest and greatest pain reduction based on both von Frey filament testing and hot plate testing. LA increased the paw withdrawal force threshold (WFT) from 0.437 g ± 0.07 (at 9 weeks) to 0.916 ± 0.65 g WFT (at 10 weeks) post-PMM in von Frey tests (Figure 1A). Retention times in hot plate tests increased from 3 to 5 s (Figure 1B). Moderate pain relief was observed with BB (0.45 ± 0.07 g to 0.63 ± 0.14 g, 3.31 ± 0.24 to 4.03 ± 0.41 s), while BS and LR did not provide significant pain protection. We prioritized LA as the most promising therapeutic candidate. We focused on its preventive efficacy in the inflammatory stage rather than in a later stage in which the disease may be too advanced to reverse OA processes. Oral gavage treatment with LA twice weekly was started at the time of joint injury. It significantly reduced pain, as shown in mechanical allodynia by von Frey filament testing (Figure 1C) and hot plate testing (Figure 1D). Paw WFT improved from 2.15 ± 0.30 g to 2.6 ±0.90 g after only three administrations of LA at 2 weeks, and reached its maximal strength by 5 weeks and lasted for 12 weeks post-PMM. LA also significantly improved heat tolerance (70% of no pain level).

### 3.2. LA Treatment Not Only Rapidly Reduced Pain but Also Prevented Cartilage Degradation

**Figure 2:**
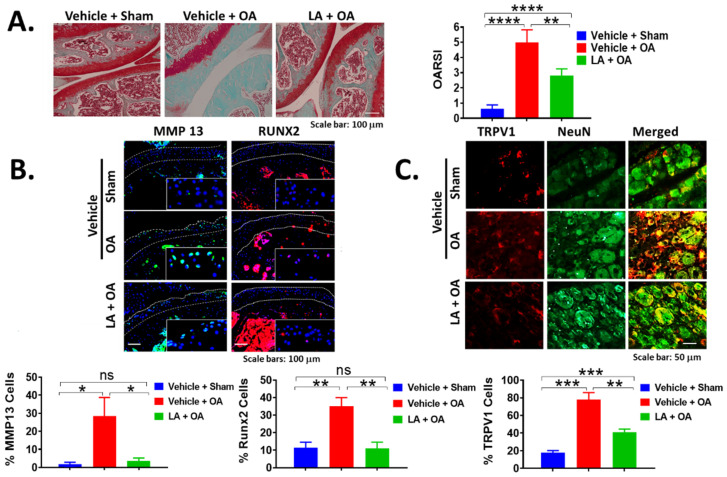
(**A**) LA treatment protects joints from cartilage damage and reduces the levels of MMP-13 and RUNX2 in cartilage, and TRPV1 in DRG sensory neurons. Safranin-O staining of OA knee joint treated with either LA or vehicle (twice/week) for 12 weeks compared to the sham group. Scale bar: 100 μm (left panel). The degree and severity of damage in articular cartilage were measured using the OARSI scoring system by comparing an LA-treated group (LA + OA, N = 6 mice) with a vehicle group (Vehicle + OA, N = 4 mice) (right panel). Data are expressed as mean ± S.E.M. Statistical analyses were carried out using 2-way ANOVA followed by Tukey’s multiple comparison analyses. The vehicle treatment group was compared with an LA group. Significance: ** *p* < 0.01 and **** *p* < 0.0001. (**B**) LA treatment reduces MMP13 and RUNX2 in cartilage and TRPV1in DRG. Immunofluorescence staining for MMP13, a potent collagenase, and RUNX2, a marker of hypertrophic chondrocytes in knee joints (Scale bars: 100 μm), and (**C**) TRPV1, a nociception marker the DRG sensory neurons (Scale bar: 50 μm). The intensity of immunofluorescence staining is plotted as % of positive cells (bottom panel). Neuronal nuclei were counterstained with NeuN to localize the DRG neurons. White arrows designate the protein of interest. Data are expressed as mean ± S.E.M. Statistical analyses were carried out using 2-way ANOVA followed by Tukey’s multiple comparison analyses. The vehicle treatment group was compared with the probiotic group. Significance: * *p* < 0.05, ** *p* < 0.01 and *** *p* < 0.001.

To assess whether LA has beneficial effects by protecting cartilage tissue, we graded histological measurement of proteoglycan depletion as visualized by Safranin-O/fast green staining of knee joints 12 weeks post-PMM (Figure 2A). We compared the Vehicle + Sham group (N = 4) with the LA-treated OA group (LA + OA, N = 6 mice) and a vehicle treated OA group (Vehicle + OA, N = 4 mice). OA knee joints showed significant protection in the LA-treated group as judged by the OARSI scoring system, which measures the degree and severity of damage. The articular cartilage surface of LA-treated mice was almost as intact as in sham surgery mice (Figure 2A left panel). OARSI scores showed protected knee joints in the LA + OA group compared to Vehicle + OA and Vehicle + Sham mice. The average OARSI score in the LA-treated group was almost half of that in the vehicle group (2.75 ± 0.4 vs. 5.0 ± 0.9 (*p* < 0.01), Figure 2A right panel). This result indicates that LA confers significant cartilage protection. Immunofluorescence microscopy of cartilage in OA mice revealed that LA treatment (LA + OA) reduces the matrix metalloproteinase 13 (MMP13), which is a potent collagenase that degrades cartilage, and the Runt-related transcription factor 2 (RUNX2), which is a critical regulator of cartilage hypertrophic markers (Figure 2B). MMP13-positive cells in cartilage increased from 2 ± 0.5% in sham surgery mice (blue bar) to 28.33 ± 10.5% in OA mice treated with vehicle (red bar), while falling back to 3.67 ± 0.7% (*p* < 0.05) in the LA-treated OA mice (green bar). Levels of RUNX2-positive hypertrophic chondrocytes increased from 10 ± 3.4% (blue bar) to 35 ± 3.6% in OA with the vehicle group (red bar) and were reduced to 10.6 ± 4.4% upon LA treatment (*p* < 0.01, green bar, Figure 2B bottom panels). Thus, our results indicate that LA treatment protects knee joints from pathological progression of OA, at least in part, by reducing MMP13 and its upstream regulator, RUNX2. Using immunofluorescence microscopy, we examined dorsal root ganglia (DRG) of LA-treated mice for a nociception marker, transient receptor potential cation channel subfamily V member 1 (TRPV1), which promotes a sensation of heat, pain and noxious environmental stimuli in primary afferent sensory neurons (Figure 2C). The neuron-specific nuclear protein NeuN was used as a counterstain to visualize the neurons in DRG. TRPV1 was significantly reduced from 78.33 ± 4.9% to 42 ± 1.5% in LA-treated mice (*p* < 0.01), consistent with the strong pain reduction in hot plate tests (Figure 1B,D). Collectively, our results indicate that LA acts as an effective disease modifier when treatment is started at the time of joint injury (inflammatory joint pain stage).

### 3.3. LA Treatment in Chronic OA Altered the Expression of Pain Markers, Neurotrophic Factors and Pro-Inflammatory Cytokines in the Distal Colon of OA Mice

**Figure 3:**
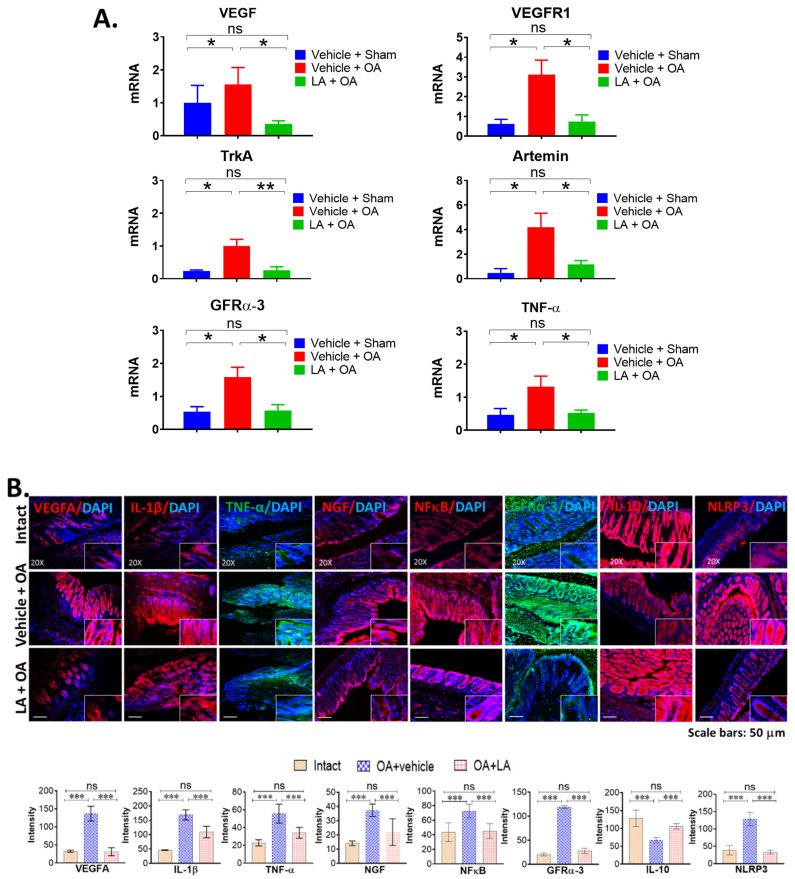
(**A**) LA treatment altered the mRNA expression of pain markers, neurotrophic factors and inflammatory cytokines in distal colon. mRNA expression by real-time quantitative polymerase chain reaction (RT-qPCR) analyses of vascular endothelial growth factor (*Vegf*), and VEGF receptor 1 (*Vegfr1*), TrkA (*Ntrk1*), artemin (*Artn*), a neurotrophic factor from the glial cell-derived neurotrophic factor (GDNF) family ligands, its receptor GFRα-3 (*Gfra3*), and TNF-α (*Tnf*) in the distal colon of mice treated with LA or vehicle twice/week for 12 weeks after OA induction. RNA isolation from distal colons of LA-treated mice was performed, followed by cDNA syntheses, and qPCR was performed targeting *Vegf*, *Vegfr1*, *Ntrk1*, *Artn*, *Gfra3* and *Tnf* gene expression. Data are expressed as mean ± S.E.M. Statistical analyses were carried out using 2-way ANOVA followed by Tukey’s multiple comparison analysis. The vehicle treatment group in sham surgery (N = 3) and PMM surgery group (Vehicle + OA, N = 3) are compared with the probiotic group (LA + OA, N = 3). RT-qPCR was repeated three times. Significance: * *p* < 0.05 and ** *p* < 0.01. (**B**) Immunofluorescence staining of the distal colon in LA-treated OA mice. Expression of inflammatory cytokines IL-1β, TNF-α, NFκB and pain markers, including VEGFA and GFRα-3 in the distal colon of mice treated with LA (LA + OA) was significantly reduced, except for the anti-inflammatory marker IL-10 (which was increased), compared to the control group (Vehicle + OA). The top panel shows IF staining of each factor merged with DAPI staining. The lower panel shows the intensity quantification. Data are expressed as mean ± S.E.M. N = 4 each of Intact, OA + Vehicle, OA + LA. Statistical analyses were carried out using 2-way ANOVA followed by Tukey’s multiple comparison analyses. The vehicle treatment group was compared with the probiotic group. Significance: *** *p* < 0.001.

Gene expression of several pain markers was analyzed by RT-qPCR in the distal colon (DC) from 3 Vehicle + Sham group, 3 Vehicle + OA group and 3 LA + OA group mice (Figure 3A), the most common location of inflammation such as in ulcerative colitis. We monitored *Vegfa*, *Vegfr1* and TrkA (*Ntrk1*), a cognate receptor for nerve growth factor (*Ngf*). We also examined artemin (*Artn*), a GDNF-like neurotrophic ligand, and its receptor, GFRα-3 (*Gfra3*), and the pro-inflammatory cytokine tumor necrosis factor alpha (TNF-α/*Tnf*) that together affect both joint pathology and neuro-inflammation. LA treatment significantly reduced mRNA levels of *Vegfa* (1.56 ± 0.5 to 0.37 ± 0.1, *p* < 0.05), *Vegfr1* (3.11 ± 0.81 to 0.75 ± 0.77, *p* < 0.05), *Ntrk1* (1.0 ± 0.2 to 0.29 ± 0.1, *p* < 0.01), *Artn* (4.21 ± 1.28 to 1.15 ± 0.24, *p* < 0.05), *Gfra3* (1.59 ± 0.39 to 0.65 ± 0.2, *p* < 0.05), and *Tnf* (1.33 ± 0.34 to 0.53 ± 0.05, *p* < 0.09) by 60 to 80% in DC. These pain marker reductions in DC are entirely consistent with the protective effects of LA on chronic OA pain. Further, this pain reduction mediated through alteration of the gastrointestinal microbiota is novel. Immunofluorescence microscopy of DC sections (N = 4 each of Intact, OA + Vehicle, OA + LA groups) showed expression of pro-inflammatory factors (IL-1β, TNF-α, NFκ-B) and anti-inflammatory factor IL-10 as well as several pain mediators, including VEGFA, GFRα-3, NGF and the inflammasome NLRP3 (Figure 3B). Surgical OA increased all tested inflammatory factors and pain markers in the DC (comparing Vehicle + OA with intact sham) by 2 to 3 fold, while levels in LA-treated mice were similar to those in controls (comparing LA + OA with Vehicle + OA). Conversely, OA mice exhibited levels of anti-inflammatory cytokine IL-10 decreased by 50%, while LA reversed IL-10 levels to near those in the intact controls. Therefore, these results validate the concept that LA suppresses inflammatory processes in the DC in mice with experimentally induced OA.

### 3.4. LA Reduced Spinal Glial Activity and Inflammatory Markers in Spinal Cords of OA Mice

**Figure 4:**
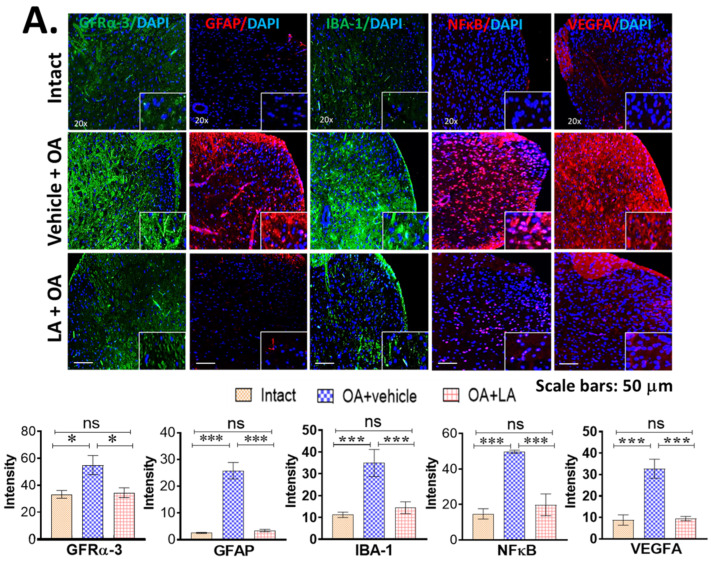

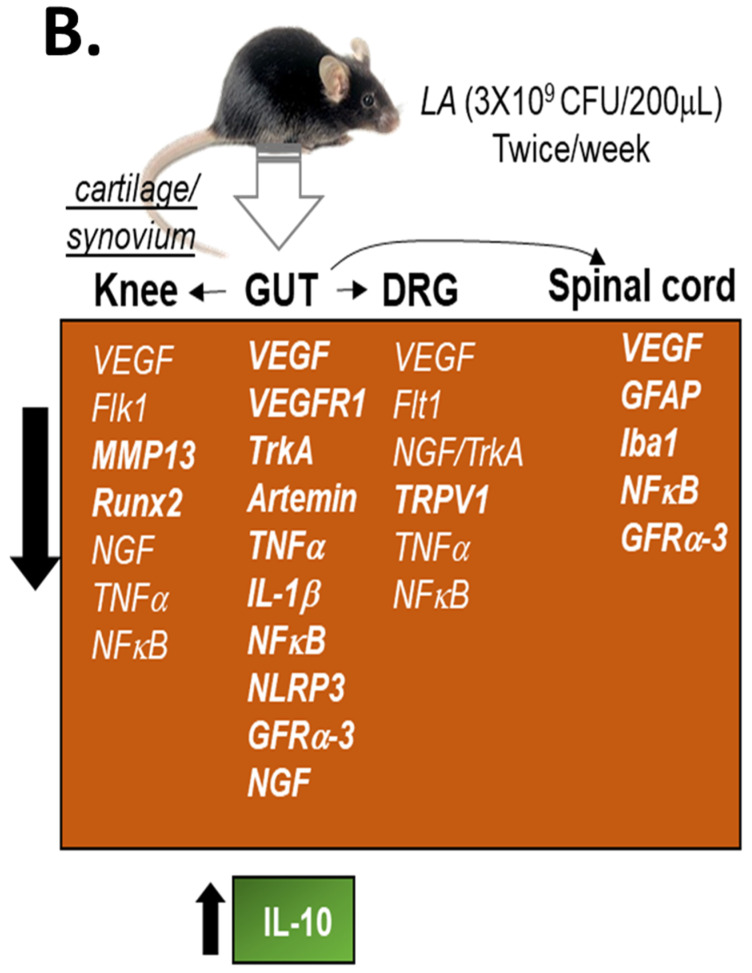
(**A**). LA treatment reduced spinal glial activity and inflammatory cytokines in SC. Expression of GFRα-3, GFAP, NFκB, VEGFA, IBA-1 in spinal cords of mice treated with LA (LA + OA) showed significant reduction compared to the control group (Vehicle + OA). Top panel shows immunofluorescence staining of each factor merged with DAPI staining. The lower panel shows the intensity quantification. N = 4 each of Intact, OA + Vehicle, OA + LA per group. Data are expressed as mean ± S.E.M. Statistical analyses were carried out using 2-way ANOVA followed by Tukey’s multiple comparison analyses. The vehicle treatment group (OA + Vehicle) is compared with the probiotic group (OA + LA). Significance: * *p* < 0.05 and *** *p* < 0.001. (**B**). Systemic impact of LA on periphery. Summary of immunofluorescence staining comparisons of the indicated tissues with or without LA treatment for 12 weeks in OA animals. Downregulation of pro-inflammatory cytokines and pain molecules and upregulation of anti-inflammatory cytokine IL-10 in peripheral tissues are summarized. Results listed here are the targets that show significant difference as judged by *p* < 0.05 (comparisons between Vehicle + OA and LA + OA group). Oral administration of LA in 3 × 10^9^ CFU markedly reduced pro-inflammatory cytokines and pain mediators in the whole body, suggesting its systemic impact.

We investigated whether LA alters cellular responses in the gut, where LA directly interacts with the host, or also in peripheral tissues. We examined N = 4 each of Intact, OA + Vehicle and OA + LA group by immunostaining of DC sections. LA significantly reduced glial fibrillary acidic protein (GFAP), an astroglial activity marker, from 25.8 ± 3 to 3.4 ± 0.3 *p* < 0.001, ionized calcium binding adaptor molecule 1 (IBA-1), a microglial activity marker (35.0 ± 6.7 to 14.5 ± 2.5, *p* < 0.001), and pro-inflammatory markers including NFκ-B (49.7 ± 0.5 to 19.8 ± 4.8, *p* < 0.001), VEGFA (32.7 ± 3.4 to 9.5 ± 0.7, *p* < 0.001), and GFRα-3, a receptor of artemin that plays a role in OA-associated chronic joint pain (53.73 ± 12.1 to 34.06 ± 4.2, *p* < 0.05), in ipsilateral spinal cords of mice (comparing LA + OA vs. Vehicle + OA) (Figure 4). GFAP is the main intermediate filament protein in mature astrocytes and an important component of the cytoskeleton in astrocytes. Microglial stress can be indexed by the microglial marker IBA-1. Merged immunofluorescence images of each factor with DAPI staining were compared to permit quantification of signal intensities. As anticipated, expression of GFRα-3, GFAP, NFκ-B, VEGFA, and IBA-1 increased in SCs of OA mice compared to control, but LA treatment reduced this to control levels (Figure 4A).

The close links between oral LA treatment and systematic impact are illustrated by reduced expression of inflammatory factors and pain mediators in peripheral tissues (Figure 4B). Oral administration of LA (3 × 10^9^ CFU for 12 weeks) after surgical induction of OA downregulated pro-inflammatory and pain-related factors but upregulated the anti-inflammatory cytokine IL-10 in peripheral tissues (only significant differences with *p* < 0.05 between Vehicle + OA and LA + OA groups are shown).

These results show that oral administration of LA has a systemic impact and reduces inflammatory cytokines and pain mediators throughout the whole body.

### 3.5. Oral Administration of LA Significantly Altered the Fecal Microbiome in OA Mice

**Figure 5:**
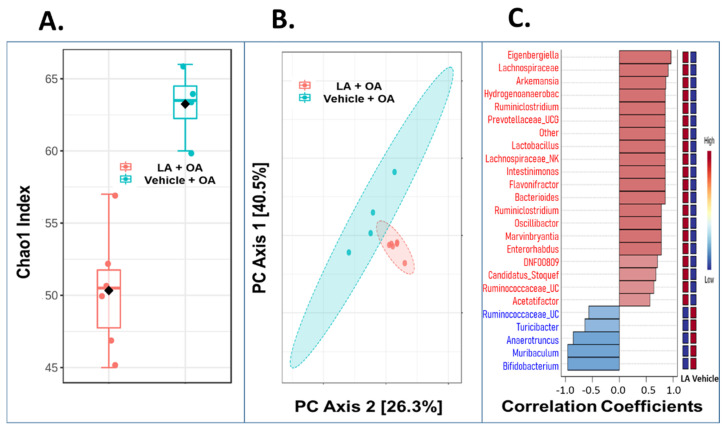
(**A**) Effect of LA treatment on mouse fecal microbiota. Alpha diversity (Chao1 index) was significantly higher in the vehicle group relative to LA-treated animals (*t*-test *p* < 0.0003). (**B**) Principal coordinates analysis (PCoA) of total microbial community structure at the taxonomic level of species/feature. Permutational MANOVA (PERMANOVA) analysis demonstrated significantly different microbial community structure between treatments (F-value: 8.4961; R-squared: 0.51504; *p*-value < 0.009). (**C**) Dominant differentially abundant taxa between the two treatment groups based on Spearman rank correlation at the taxonomic level of genus.

Therapeutic effects on OA pain and joint pathology by oral administration of LA may involve changes in the gut microbiome. Therefore, we characterized the fecal microbiome profiles as a proxy for the gastrointestinal (GI) tract microbiome using 16S rRNA gene amplicon sequencing in LA-treated (N = 6) vs. vehicle-treated OA mice (N = 4) (Figure 5). Fecal microbial alpha diversity (as assessed by the Chao1 index) was significantly higher in the vehicle group relative to LA-treated animals (*t*-test *p* < 0.0003) (Figure 5A). Principal coordinates analysis (PCoA) and PERMANOVA analysis revealed significant differences in total microbial community structure at the taxonomic level of genus (Figure 5B) as well as prominent differentially abundant taxa between the two groups based on Spearman rank correlation at the taxonomic level of genus (Figure 5C). LA treatment showed a significant enrichment in the genus Akkermansia muciniphila (0.7–6.2% of total community), a beneficial commensal microbe that colonizes the mucosal layer of the gut, which was largely absent in the vehicle group (0–0.08% of the total community; DESeq2 q = 8.20 × 10^−6^). Bacteria from the family Lachnospiraceae (NK4A136), which are prevalent obligatory anaerobic microbes in the mammalian gut, were highly abundant as expected and also significantly enriched by LA treatment (4.4–19.8% of the total community in the LA treatment vs 2.3–7.0% of the total community in the vehicle treatment; DESeq2 q = 0.01). There are taxa with higher and lower relative abundance in LA-treated mice. Hence, the results show that LA treatment significantly alters the fecal microbial community structure and lowers the microbial diversity. Importantly, LA treatment does not result in community domination, but rather alters the total structure of the microbial community by altering the population distributions of several species.

## 4. Discussion

The impact of gut microbiome dysbiosis on joint degeneration in OA is reflected by alterations in serum levels of bacterial metabolites [24]. This gut–joint axis in OA can also be significantly influenced by antibiotics or dietary supplements that generate shifts in the gut microbiome [25]. Experimental OA mice given antibiotics to induce dysbiosis showed reduced levels of systemic lipopolysaccharides and inflammatory factors that decreased MMP13 expression and improved OA symptoms [25]. Our data demonstrate that LA reverses dysbiosis in our surgical OA model. This probiotic reversal is accompanied by acute reductions in inflammatory factors (IL-1β, TNF-α, NFκ-B and NLRP3), nociceptive mediators (VEGF, NGF, artemin and GFRα-3) and catabolic enzymes (RUNX2 and MMP13) in knee joints, distal colon, SCs and DRG of LA-treated OA mice. These changes collectively alleviate OA symptoms, reflecting the functional relationships in OA between the gut–joint axis and multiple biological processes, including inflammatory responses, cartilage homeostasis and nociception.

Our results support the concept that LA may rebalance the pro-inflammatory environment by elevating the level of anti-inflammatory cytokines and lowering pro-inflammatory factors. This rebalancing may be achieved by intervening in the intracellular activation of NFκB signaling pathways, which play key roles in the inflammation and regulation of inflammasomes [26]. Consistent with the latter, our data show that LA reduces the levels of the inflammasome marker NLRP3 in distal colon.

Our findings on the benefits of probiotic therapy using LA are consistent with the pathological links between gut microbiota, adiposity-derived inflammation and metabolic OA and abundance of Lactobacillus and Methanobrevibacter species [24]. The reductive effects of LA on pain markers and the severity of OA pain may be linked to the relative abundance of Streptococcus species in the gut microbiome that produce endotoxins capable of driving inflammation in the knee joint and accelerating OA progression [24]. Furthermore, LA reduces the pain-related signaling ligand artemin and its cognate receptor GFRα-3, which together generate pain signals in response to inflammation and bone pain in rodents and dogs with naturally occurring OA [27]. LA strongly reduces pain in both mechanical allodynia and heat tolerance. The latter effects may be attributable to modulation of TRPV1, which mediates the detection of noxious environmental stimuli such as heat or pain in primary afferent sensory neurons, and blocking TRPV1 promoted sciatic nerve regeneration [28].

Thus, the beneficial effects of LA on pain may involve changes in intestinal microbial communities, systemic microbial toxins, the artemin/GFRα-3 signaling pathway and TRPV1.

Histological changes in osteo-chondral tissues within OA joints reflect modifications in cartilage homeostasis that are in part related to Runx2. Runx2 controls differentiation of mineralizing osteoblasts, but also is responsible for hypertrophic chondrocytes during OA disease progression [29] and is a key transcriptional regulator of classical hypertrophic markers such as Mmp13 and Col10a1. The pathologically elevated levels of RUNX2 and its cartilage-degrading target MMP13 were both decreased in cartilage of LA-treated OA mice. Thus, LA may have a beneficial effect in preserving joint integrity in OA by inhibiting the RUNX2/MMP13 pathway. Beyond these local effects in joint tissues, we observed suppression of angiogenesis factor VEGFA and VEGFR1 in both DC and SC combined with reduced inflammatory cytokines in peripheral tissues of LA-treated OA mice. These findings indicate close connections between oral administration of LA and its systemic impact throughout the body and effects on joint tissue homeostasis.

We found that the LA treatments significantly altered the microbial community structure in the GI tract of OA mice based on 16S rRNA gene amplicon sequencing of the fecal microbiome. LA administration significantly enriched the genus Akkermansia and bacteria from the Lachnospiraceae family. Lachnospiraceae are closely associated with beneficial anti-inflammatory effects [30]. The increased prevalence of Lachnospiraceae strongly supports the idea that LA promotes the production of short chain fatty acids (SCFAs) and anti-inflammatory factors, resulting in desensitizing peripheral sensory neurons.

The clinical implications of our study highlight the therapeutic efficacy of LA in reducing pain molecules, cartilage-degrading enzymes and inflammatory cytokines by alteration of the microbiome of the GI tract. These changes result in alleviation of OA symptoms and prevent further degeneration of OA joint integrity.

In humans, Clostridiales and the genus Streptococcus were associated with OA pain [4], and nutrient metabolism influenced functional changes in the gut microbiome in OA [5]. Our pre-clinical data showing that LA restores gut microbial balance and improves OA pain and joint pathology can be applied to human subjects. Therefore, probiotic-based approaches for OA therapy are promising. Future studies may provide further evidence for the considerable potential that LA may have in clinical translation of these findings in probiotic-mediated prevention or treatment strategies for OA. Combining LA with selective prebiotics (synbiotics) and/or fecal microbiota transplantation [8] could amplify LA efficacy against chronic joint pain control and joint integrity by modifying the gut/microbiome–OA axis.

Study limitations in our current manuscript are the relatively small sample sizes that were used for the microbiome data to characterize microbial communities. Our studies are not the first to consider LA for OA treatment, but rather provide strong corroborating evidence and substantial new findings on microbial effects that complement previous studies on the effects of LA in ameliorating pain and protecting against cartilage destruction in a chemically induced (i.e., monosodiumiodoacetate/MIA) rat model for OA that did not investigate the microbiome [15]. Another limitation is that we restricted our control groups for comparisons to reduce animal numbers, which prevented us from fully accounting for all technical parameters that could influence the results. However, these limitations do not substantially detract from the main findings of this work that LA had beneficial effects on pain, cartilage histology and molecular markers in our OA model.

## 5. Conclusions

LA rapidly reduced joint pain by desensitizing nociception, reducing the levels of pain modulators and pro-inflammatory factors, while decreasing spinal glial activities in our experimental OA animal model. LA effectively protected joint tissue integrity during OA disease progression, which was corroborated by significant alterations in the OA gut microbiome. Our study suggests that LA treatment could be developed as an excellent OADMD (with an established safety profile as probiotic) that has the potential for rapid translation to clinical settings.

## Abbreviations

ARTN	Artemin
CRP	C-Reactive Protein, DRG: Dorsal Root Ganglion
GDNF	Glial Cell-Derived Neurotrophic Factor
GFAP	Glial Fibrillary Acidic Protein
GFR α3	Glial Cell-Derived Neurotrophic Factor Family Receptor α3
IBA-1	Ionized calcium Binding Adaptor molecule-1
IF	Immuno-Fluorescence
IFN	Interferon
ILs	Interleukins
LA	*Lactobacillus acidophilus*
MIA	Monosodium Iodoacetate
MMP-13	Matrix Metalloproteinase-13
NF-κB	Nuclear Factor kappa-light-chain-enhancer of activated B cells
NGF	Nerve Growth Factor
NLRP3	Nucleotide-binding domain Leucine-rich Repeat and Pyrin domain containing receptor 3
OA	Osteoarthritis
OADMD	OA Disease Modifying Drug
OARSI	score: Osteoarthritis Research Society International score
PMM	Partial Medial Meniscectomy
PCoA	Principal Coordinates Analysis
RUNX2	Runt-related transcription factor 2
RTqPCR	Real Time Quantitative Polymerase Chain Reaction
SC	Spinal Cord, SCFA: Short-Chain Fatty Acid
TrkA	Tropomyosin Receptor Kinase A
